# Perceptions of Pediatric Nurse Practitioners and How a Pulmonary Function Printout Influenced Practice

**DOI:** 10.1155/2012/794585

**Published:** 2012-08-28

**Authors:** Susan Gresko, Elizabeth Burgess Dowdell

**Affiliations:** ^1^Department of Nursing, Temple University, Philadelphia, PA 19122, USA; ^2^College of Nursing, Villanova University, Driscoll Hall, 800 Lancaster Avenue, Villanova, PA 19085, USA

## Abstract

The rate of asthma in the pediatric population has risen over the last two decades and is now considered to be the most common serious chronic disease in children and adolescents as well as a serious public health concern. In one suburban, Pennsylvania nurse-managed clinic, a group of pediatric nurse practitioners (PNPs), noted an increase in the number of children with asthma and purchased a pulmonary function machine (Spirometer). The purpose of this paper is to discuss how the integration of a pulmonary function measurement printout influenced a small group of PNPs visit satisfaction, their delivery of nursing care, and the response of the families. As the incidence of asthma increases in the pediatric population, nurse practitioners and other healthcare professionals can take a leading role in patient teaching and provision of care by augmenting their practice with new technology combined with continued education for the client and family.

## 1. Introduction

D.G. is an 11-year-old boy who lives in a suburb located outside of a major Northeastern city where his primary care provider is a pediatric nurse practitioner (PNP) based in a nurse-managed clinic. D.G. has an extensive medical history beginning with his premature birth at 24 weeks of gestation to a teenage mother and a history of asthma. Due to social issues, D.G. was removed from his mother's care and placed into the foster care system where he has lived with the same foster mother for the past 10 years. Because of his multidimensional health needs as well as developmental difficulties, D.G. is seen in the clinic every 6 months for medical and medication management. During his last visit, the PNP noted on history that after running around outside or following gym class D.G. shared “my chests hurts and I sound yucky when I breathe.” The PNP decided to have D.G. to use the clinic's newly purchased pulmonary function machine (Spirometer) as part of his assessment. Although D.G's physical exam showed clear auscultation breath sounds bilaterally with no work-of-breathing and brisk capillary refill, the Spirometer recorded that D.G. had “mild pulmonary restriction.” Using this finding, the PNP reevaluated as well as changed D.G.'s medications to continue his Singular dosage, increased his Flovent inhaler from daily to twice a day (BID), and booked a follow-up visit for 2 weeks. At the follow-up visit D.G. repeated having the same respiratory difficulties without relief, with the same finding of “mild pulmonary restriction” recorded on the Spirometer. The PNP then specifically asked D.G. and his foster mother about his asthma medications. The foster mother said after much discussion that she was giving D.G. his “asthma nebulizer of Albuterol (Ventolin) twice a day now to cover him in the morning and in the evening” with only “occasional use of the Flovent inhaler.” At this point, the PNP showed the Spirometer printout to reinforce that D.G. needed his medications on a regular basis. The PNP discussed and demonstrated the proper use of the inhaler in addition to how and when to use the Albuterol nebulizer as a rescue medication. At the next follow-up visit 2 weeks later, D.G. reported “nothing is happening, all's good” which was supported by the Spriometer which recorded a finding of “normal pulmonary function and spirometry.” The foster mother shared that she had been giving D.G. his Flovent inhaler twice a day and the Singular at night, with only two uses of the Albuterol nebulizer. The PNP reinforced this medication administration behavior by showing D.G.'s printout and comparing it to the previous printout. Both D.G. and his foster mother were surprised at how “good he has gotten since the last visit.” At the end of this visit, the PNP shared with colleagues the case and how using the printout had changed her practice with this client and his foster mother.

Asthma is a significant and growing public health problem in the United States, with the Centers for Disease Control and Prevention estimating that about 7 million children under age of 18 years (5% to 15% of the pediatric population) are affected [1, 2]. Asthma is considered the most common serious chronic disease in children accounting for approximately 3 million visits to health care providers and over 200,000 hospitalizations annually [1, 3]. Asthma is characterized by chronic inflammation of the airways that results in swelling, bronchoconstriction, and excess mucus production that impedes airflow. In children, this chronic condition has the potential to remodel the airway causing significant loss of pulmonary function [4–6]. Pennsylvania, in particular, has one of the worst state rates for asthma admissions in the United States. The national average rate of pediatric asthma hospital admissions is 69.9 per 100,000, whereas in Pennsylvania hospital admissions were found to be almost triple at 268.8 per 100,000 [7]. Recent research suggests that the prevalence rates of asthma in children are on the rise with nearly a 72% increase in cases reported between 1982 and 1994 [3], making pediatric asthma the third leading cause of hospitalizations in persons under the age of 18 years. As asthma rates increase so do the asthma-related direct and indirect health care costs. It is estimated that the US spends approximately $3.2 billion per year on direct care costs to treat asthma [3]. Indirect costs, including nonmedical economic losses due to premature death, days missed from work, caregiver costs, travel, and waiting time, and losses that correspond to approximately 14 million days of missed school, account for an additional $726 million [3] in economic losses and costs.

Risk factors for asthma include family history, premature birth, viral infections, allergen exposure, and exposure to irritants such as tobacco smoke [4, 6]. The course of asthma may vary in the pediatric population with young children, school-age children, and adolescents being managed and managing their asthma and related symptoms differently. Typically younger children rely on a parent or caregiver to monitor their health status, coordinate visits to healthcare providers, and manage medications, including rescue drugs (inhalers, nebulizers, etc.). As children age grows into adolescence, expectations change such that the monitoring and management of asthma and symptoms are increasingly taken on by the individual adolescent or young adult.

In caring for a child who has asthma a physical examination is an essential component in the routine, follow-up care, and management. In addition to a complete health history and physical assessment, the use of pulmonary function testing is also becoming important in providing a complete clinical picture of how an asthmatic is functioning. Spirometry is one type of pulmonary function measurement that is being used more frequently by primary care providers in the community setting. The Spirometer device is a piece of equipment that is used for measuring the volume of air inspired and expired by the lungs [8]. To use the Spirometer an individual is asked to take a deep breath, and then blow out as hard and as fast as possible using a mouthpiece connected to the machine with tubing. The Spirometer measures the total amount exhaled by an individual, called the forced vital capacity (FVC), and how much was exhaled within the first second. Normal spirometry results are based on the age, height, gender, and ethnicity of the person being tested [8]. The results are calibrated immediately by the Spirometer and are recorded on a colored graph printout along with the expected normal values for the individual. Results are considered abnormal if the result is less than 80% of the predicted value. In an asthma population spirometry can be used when asthma symptoms are present and can assist the provider in the documentation of normal or near-normal airway function. The current medical literature suggests that a spirometry measurement taken during follow-up visits helps to assess the maintenance of airway function and the long-term effectiveness of pharmacotherapy [8]. The assessment should include monitoring of asthma signs and symptoms and periodic pulmonary function, and tracking of medications and asthma exacerbations.

As the number of children with asthma increases so has the role of the advanced practice nurse caring for them in a community setting. In one suburban, Pennsylvania nurse managed clinic, a group of pediatric nurse practitioners (PNPs), noted an increase in the number of children with asthma as a primary diagnosis. As a result this clinic purchased a pulmonary function machine to use with their pediatric asthma population. A project was designed and implemented using a sample population consisting of multicultural families with a low socioeconomic mean income that had a child between the ages 6 to 18 years with a diagnosis of asthma. The purpose of this paper is to discuss how the integration of a pulmonary function measurement printout influenced a small group of PNPs satisfaction with the client visit, their nursing care, and the response of the families.

## 2. Methods

### 2.1. Subjects

The sample size for this qualitative research project consisted of five primary care PNPs who received spirometry training specific to the machine purchased by the nurse managed community clinic where the PNPs practiced. The PNP age range was 40 to 54 years old with a mean of 20 years of practice. The sample size of children involved a total of 19 children with asthma in addition to 16 parents or guardians. The child sample consisted of 11 boys (58%) and 8 girls (42%). The adult samples were all women, 12 biological mothers, 1 biological grandmother, 1 biological great-grandmother, and 2 foster mothers. The mean age for the boys was 12.1 years (age range 6 to 18 years) and 11.2 years for the girls (range 6 to 16 years). For the total child and adult population 26 (68%) were *African American, *6 (16%) *Caucasian*, and 6 (16%) *Hispanic* (Table 1). The children were asked to rate their asthma as being mild, moderate, or severe. The majority of children 10 (53%) rated their asthma as “mild,” 8 (42%) reported “moderate,” and only 1 rated himself as “severe.” The most common asthma symptoms reported by the children were wheezing (47.4%), coughing (36%), shortness of breath (32%), and chest tightness (31%).

Of the pediatric sample the majority (*n* = 14; 73.7%) reported having a family history of asthma with the majority (58%) that reported at least one sibling with asthma. Over half (53%) of the children with asthma reported having at least one allergy and over half (55.6%) reported having sensitivity to environmental allergens. The majority of children (84.2%) stated that changes in the weather also affected their asthma symptoms. Overall, this sample of children with asthma was well managed on their medications and had minimal hospital visits for asthma. All but one child (95%) reported taking daily asthma medication, all reported having medication at home, and four (21%) reported use of home remedies (e.g., herbal mixtures, poultices). Six (32%) reported that they received asthma medication while at school, five (26%) denied school medication use, and 8 had missing data. When asked “*Do you believe these (daily) medications have helped with your asthma*” the majority of children (16; 85%) said “*no,*” 1 said “*yes,*” and 2 were missing data.

Prior to the beginning of data collection Institutional Review Board (IRB) approval from the Visiting Nurse Association (VNA) and the second author's institution was obtained. For all participants the details of the study were discussed prior to the beginning of the clinic visit. Information provided to each parent or guardian, and child included informed consent, child assent, patient and family confidentiality, the nature of the questions to be asked in the study, in addition to details regarding the pulmonary function measurement (spirometry), and an explanation of the Spirometer. An information sheet describing the purpose of the study and an informed consent was given to the parent by the PNP. The children and adolescents who participated were also given an information sheet by the PNP in addition to a verbal explanation of the study, what would happen during the clinic visit as well as an introduction to the Spirometer. Only parents or guardians, who provided written consent in addition to children who gave their assent or adolescents who gave both assent and written consent, were allowed to participate in the study.

### 2.2. Setting

Data collection took place in a nurse managed community clinic located in a suburb of Philadelphia, PA, USA. The clinic provides family-oriented primary care services which include well child visits, sick child visits, medication intervention, and case management services. Bachelors-prepared registered nurses and master's prepared PNPs provide services to the children, mothers, and families.

### 2.3. Procedure

The original intention of this project was to recruit a robust sample of children with asthma and parents or guardians from two clinics with multiple PNPs providers to make home visits. However, recruitment of subjects for a home visit proved to be exceptionally difficult. It is important to note that on the initial telephone call every parent contacted agreed to participate in the study. Difficulty was encountered on the confirmation call the night before the scheduled home visit or with the call on the day of the visit. Many of the families in the time between phone calls, which was no longer than 21 days, had disconnected phone numbers, nonworking phone numbers, had moved, or placed restrictions on the home visit that could not be accommodated by the PNP. For example, two families had moved in the two weeks between the first phone call and the confirmation phone call, six families had disconnected phones in the same time period, and one mother agreed to the home visit but stated that the visit could “only happen when TJ (the male partner) was not at home” further stipulating that the visit could “only last for 30 minutes on Friday at 7 AM.”

At the end of 8 months only 4 home visits had been completed and the decision was made by the project team to recruit at one clinic location during a pediatric sick or well visit. By eliminating the home visit and using the primary clinic as the site we were able to have access to both child and parent or guardian. It is important to note that doing data collection during the clinic visit increased the average clinic visit time of 15 minutes to almost an hour which became a factor that affected the number of families willing to participate.

### 2.4. Data Management and Analysis

All PNPs involved in this project were asked (1) to identify which agency clinical asthma pathway was used with the client, (2) to complete a one-page sociodemographic sheet on the child, and (3) to keep detailed notes of their experience with the Spirometer. These notes frequently contained a detailed description of the client's clinic visit, the PNP's assessment of the visit, her thoughts specific to using the Spirometer. Although the parent or guardian and the child did not have any instruments to complete, all were asked to share their thoughts, both verbal and written, with the PNP during and at the end of the visit. Their comments were then included as part of the PNP notes section.

Descriptive statistics on the total pediatric data set were used in analysis with no additional methods employed due to the small sample size; however, qualitative data collected from the families in terms of notes taken by the PNP with quotes and the notes from the five PNPs who conducted the visits at the clinic were used as the basis for this qualitative project. Data analysis was a continuous process that involved the principal investigator (PI) transcribing the data from the PNP notes. The transcriptions were then read for content by PI and co-PI of the project. All client information (name, family name, medical chart number, and address) was removed from the clinical pathway and sociodemographic sheet. Additionally, each PNP was assigned a number which was then used on the clients file to help tracking which PNP provided care to which client.

### 2.5. Use of Spirometry

Use of a pulmonary function machine, a Spirometer, occurred in all of the clinic visits. Specifically, the child participant blew into a hand held machine that produced a graphic printout displaying numerical values that provided a measurement. The printout result recorded the child's pulmonary function on a graph with his or her function compared to what would be considered normal for his or her height, weight, and age. Each of the 19 subjects seen in the clinic used a Spirometer following the PNPs physical assessment. The majority (14) of children had a spirometry reading of “normal pulmonary function,” four produced a reading of “mild pulmonary restriction,” and 1 read “moderate pulmonary restriction.” Clinic policy of best practice had that any client with a mild, moderate, or severe pulmonary spirometry reading be treated according to the clinic's asthma clinical pathway. Treatment plans on this pathway included medication reassessment with the introduction of new meds or higher dosages, radiology films, return visits within a specific time period, or a visit to the emergency room for further evaluation. In every case the PNPs, based on the pulmonary function reading, followed the appropriate clinical pathway. In this study, due to the small sample size the spirometry readings were not tabulated, analyzed, or coded for statistical inference.

## 3. Results and Discussion

### 3.1. Using the Spirometer Printout and Higher Visit Satisfaction

Nursing practice in the 21st century is being influenced and changed by the technology that has become an integral part of everyday life (e.g., the Internet, clinical simulations, Webcasts, and Podcasts). In our study, technology was defined as incorporating the use of a pulmonary function machine into practice. Although there are no significant findings to report from the Spirometer itself, incorporation of the graph printout of the child's pulmonary function was found to be a repetitive theme in the PNPs notes. The printouts produced by the machine were not shown or shared with every family but when the printout was used the nurses' notes showed a difference. Those using the printout(s) during the visit, specifically in their teaching, reported receiving positive feedback from child and parent or guardian along with higher levels of visit satisfaction. For example, one PNP documented that after using the printout with a 10-year-old girl the client asked “How do I improve my numbers and that line? … I want to have better numbers so my line looks right (closer to the normal function graph line).” The PNP wrote in her notes that she felt the printout of the girl's mild restriction pulmonary function specifically lead to a “longer conversation about her use of daily medications, her asthma management at home, and how best to manage her asthma at school and when out with friends.” Using the printout provided the PNP with an individualized tool to reinforce asthma teaching with both child and mother. The Institute of Medicine [9] has linked the concepts of client safety and quality of care together with a dependence upon the ability of the health care professional(s) ability to access and use advanced technology. Indirectly, the use of the Spirometer in this study influenced the practice of each PNP because it provided tangible evidence of the child's function via graph printout, but also offered the PNP a tool to use with both child and parent to reinforce asthma teaching, medication use, along with wellness choices that can promote a healthy lifestyle.

### 3.2. Using Spirometer and Higher PNP Satisfaction

Using technology to enhance patient education is one common strategy used for increasing levels of knowledge and satisfaction. Providers that can adapt to changing populations, changing conditions, and identify opportunities for health improvement in high-risk groups can also use technology in addressing their client's needs [10]. Satisfaction with the visit by all parties (child, parent, and nurse) can be an achievable goal. In our study, the PNPs that used the Spirometer reported feeling that the pulmonary function machine helped them in their practice as a tool that leads to more teaching time in addition to higher levels of satisfaction associated with the visit. The use of the graphic printout in demonstrating pulmonary function during a clinic visit was identified as an important piece of the assessment for these PNPs and one that they recommended to others within the agency as the graph provides immediate visual documentation of function. In the literature, spirometry has been used mostly for diagnostic and monitoring purposes and seldom for screening purposes [11], but in this study it also provided the PNPs with teaching moments and greater visit satisfaction. As one PNP stated, “Having an objective tool to use (the printout) that I can show patients and families helped to reinforce the asthma teaching I was providing. I believe that I was listened to and that the extra time with teaching made a difference to the child but also to my practice … I feel better about what I am doing.” The theme of being heard by child and parent after using the printout was a repetitive point made by all five PNPs. The combined variables of utilizing a machine to measure pulmonary function, using the printout to individualize the teaching combined with more teaching time and feedback from child and family, reinforced the PNPs sense of being heard which then influenced their level of visit satisfaction.

### 3.3. Patient Education Improved with Spirometer Printout Use for PNPs

**Figure 1:**
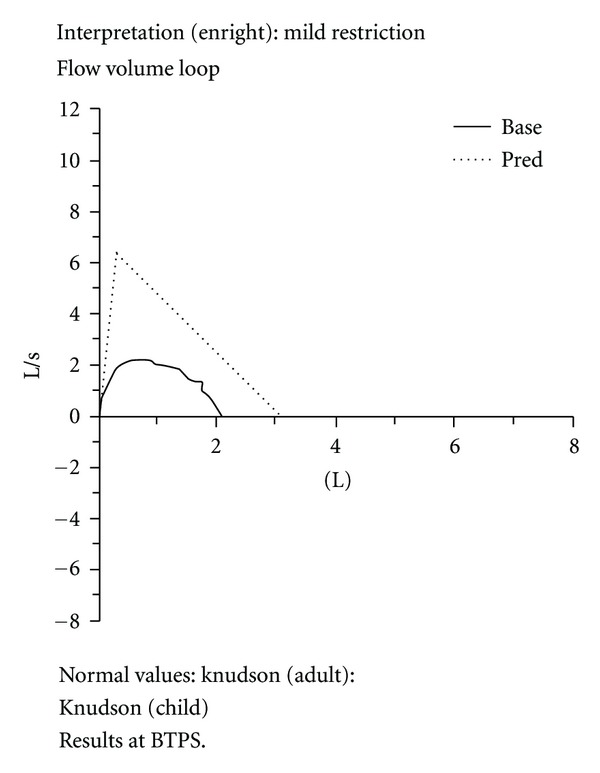


Patient education is an important part of every visit and PNPs have a reputation for being excellent teachers. For the PNPs in this study using the printout with child and parent provided a tool that could help to change patient behavior. In the case of a 16-year-old boy who was well known to the clinic staff both the teen and the PNP found using the printout to be valuable. This teen came into the clinic for a sick visit complaining of “waking up coughing every night for 2 weeks.” The PNP documented that the teen thought his “coughing all night was normal” with asthma and had not reported it to his parent or taken any of his rescue medications. The PNP had the teen use the Spirometer which showed that the teen's pulmonary function level to be significantly below the expected normal level. The teen's pulmonary line, based on the values assigned by the machine based on his expiration, was recorded as “mild restriction” (Figure 1). As a result, the PNP and teen began a discussion about his level of asthma knowledge and management using the teen's printout as the tool to begin the dialogue. At the end of the visit the teen wrote that he liked “the printout ‘cause it showed me how my asthma is doing, and I'm not doing good.” The printout also lead to the teen disclosing that he was not compliant with his daily oral or inhaled medications stating that he felt “like they just do not work for me … I hate having to carry around that inhaler, it's a pain and I get funny looks.” He also shared that he did not understand the purpose or use of his rescue medications.

**Figure 2:**
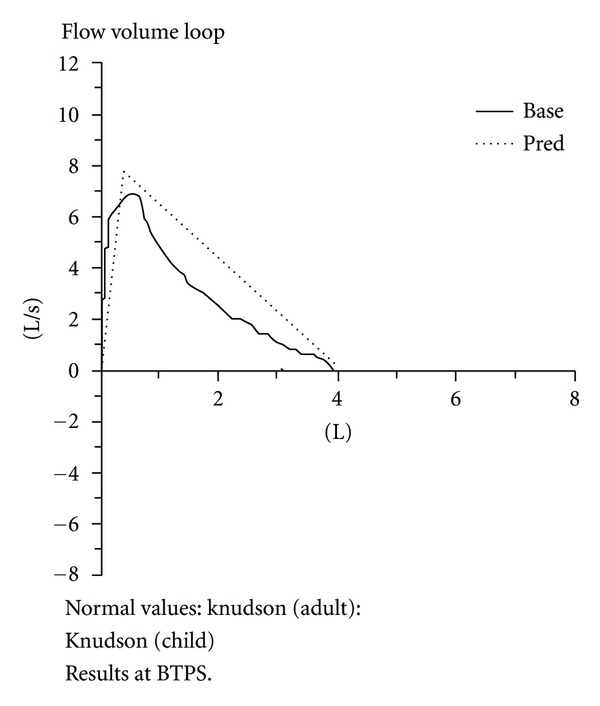


Using the printout as a starting point, the PNP spent over 20 minutes talking to the teen about his asthma, why asthma medications are prescribed, how the meds work, and the benefit(s) of daily use. She also spent time problem solving with the teen about how to better manage his medications when away from home, at school, with friends, or at work. After receiving asthma management of teaching and adjustment of his medications, he reported at his 10-day follow-up visit that he was now “able to sleep through the night … I feel like a new person with much more energy ‘cause I'm using my meds the right way.” At the follow-up visit he used the Spirometer again with better results. This time his pulmonary function values were all higher and the line denoting pulmonary function was closer in form to the normal value line (Figure 2). This visualization of the values and the graph line difference from the previous visit to the current visit had an impact on the teen that he had not experienced before and he shared that he “liked that paper (printout) ‘cause it shows me how I'm breathing.”

Simple educational interventions are being shown to improve patients' understanding and recall of information about chronic health conditions [12–14]. Utilizing teaching strategies that are concrete, individualized, and viewed as valuable by an individual may have higher success rates. Our study suggests that using the printout as part of the respiratory assessment presented the PNP a tool that was then shown as proof of function and used to open dialogue about asthma, medication use, and lifestyle choices with not only the child, but also with the parent or guardian. The PNPs in this study frequently documented that their use of the printout with the child often encouraged the parent or guardian to verbalize their own questions or concerns. For example, a 6-year-old boy stated “I want to make the line (his graph line) do better after every blow.” The mother of the boy shared at the end of the visit that she “really liked seeing him doing the blowing because the nurse (PNP) explained the test and printout to me … Now I understand why it was so important for RJ (her son) to use his medications. The pills and breathing sprays prevent an asthma attack.” In this case, because the parent and the boy agreed to be in the study the PNP used the Spirometer which recorded an abnormal pulmonary printout of the boy who was not exhibiting clinical exacerbation signs and symptoms on exam. In the notes from this case, the PNP recorded two points, the first was that the Spirometer helped to predict the potential for RJ to have an exacerbation even though the PNP had auscultated clear breath sounds bilaterally and second the extra teaching time was beneficial to the family. As a result, the boy's medications were adjusted by adding a daily inhaler, continuing his oral medication, encouraging the boy not to have the dog sleep on his bed at night, along with the purchase of pillows made of hypoallergenic materials which could aid in the prevention of an exacerbation. On a 7-day follow-up telephone call with the mother she shared that not only had RJ avoided an “asthma attack” but he was “using his medications without problem … and I feel like I know something I can do to help him.” This increase in knowledge by a parent was noted repeatedly and may be reflective that providing education alone is not sufficient to change adherence behaviors and sustain treatment adherence over time [15]. In our study, the PNPs discussed education along with medication uses using a visual printout specific to that child which over time may have provided interventions to the family's particular needs thereby encouraging better asthma management behaviors.

### 3.4. Unexpected Finding

It is important to note that there was a surprise finding from this study, in that the majority of the children who participated reported that they do not believe that their asthma medication(s) has helped them. Although it is not clear whether they were referring to a specific medication or method of administration, the finding has important implications for medication adherence in this vulnerable population. It is not uncommon for young children to have a parent managing their medications, but as a child grows into adolescence, expectations change such that the monitoring and management of asthma and symptoms are increasingly taken on by the individual adolescent or young adult. At the same time that adolescents may be assuming greater responsibility and control for their own asthma management, they are also being faced with the potential temptation to change their adherence behaviors or to behave as their peers or adults by beginning to engage in health-related risk behaviors, such as tobacco and alcohol use. Nurse practitioners are in an ideal position to promote medication adherence and symptom management. In this study, the PNPs reported that having a paper copy of pulmonary function to show as evidence of function increased their time teaching which may provide future benefits as a deterrent to risky behavior and, perhaps, as a motivator is to be symptom-free.

## 4. Conclusion

Advances in healthcare technology and everyday life have expanded the educational tools available to today's nurses, nurse practitioners, and primary care providers [16]. The major finding from this study suggests that incorporating technology (a pulmonary function machine) that provided an individualized printout graph form was beneficial to child, parent, and PNP. PNPs who used the pulmonary function machine reported feeling that their respiratory assessments were accurate when the Spirometer supported their findings or encouraged them to ask different questions of the child or parent when the printout did not support their physical assessment findings. Having the printout gave the families a visual aid and provided the PNPs with graph printouts that were used as a tool to increase teachable moments with both the child and parent. These factors supported the finding of an increased level of satisfaction by the PNP with the visit. The additional time was used most often for medication remediation, teaching about asthma medications, asthma as a disease, wellness behaviors, and healthy lifestyle choices. Short, simple interventions have been found to promote knowledge gain that can be sustained over extended time periods [14]. As the incidence of asthma increases in the pediatric population nurse practitioners can take a leading role in patient teaching and provision of care by augmenting their practice with new technology combined with continued education for the client and family. The use of a machine that provided a visual tool of the graph printout combined with the additional teaching time may promote long-term retention of new information, which may enhance later recall and thus improve patient's self-care behaviors [14]. As the findings from this study were discussed within the clinic, the PNPs, using their field notes and clinical observations, went forward to the clinic's administrative team supporting the purchase of a Spirometer for the other clinics in the VNA.

### 4.1. Recommendations

Improving health outcomes for children and adolescents is essential to achieve a healthy future for the nation [9]. Future nursing research would include a larger sample of children with asthma, parents or guardians, nurse practitioners, and nurse managed clinics. Also, using a longer timeframe would include a detailed followup to identify if the extra teaching helped to promote short-term and long-term retention of the education taught.

### 4.2. Limitations

The study findings should be viewed in light of the study limitations. Two of the most significant shortcomings in the current study relate to the small sample of children with asthma, parents or guardians, and the small number of PNPs. Specifically, with the small sample size the ability to run meaningful quantitative statistics was not possible. Additionally, the small sample size may limit the generalization of the findings to other groups. Nonetheless, the PNPs report that more teachable moments and increased visit satisfaction are valuable outcomes to nursing.

## Figures and Tables

**Table 1:** Demographics of Children and Mothers or Guardians.

Child Gender	Males	Females
	(*N* = 10) 52.6%	(*N* = 9) 47.4%
Child Age:		
Mean (SD)	12.1 (1.03)	11.2 (0.89)
Range	6–18	6–16
Child Race (*N* = 19):		
African American	90%	55%
Caucasian	0%	33%
Hispanic	10%	22%
Parent or Guardian Race (*N* = 16):		
African American	0%	76%
Caucasian	0%	18%
Hispanic	0%	6%
